# Multimodal Self-Supervised Learning for Early Alzheimer’s: Cross-Modal MRI–PET, Longitudinal Signals, and Site Invariance

**DOI:** 10.3390/diagnostics15243135

**Published:** 2025-12-09

**Authors:** Soumaya Belhaj Ali, Naglaa E. Ghannam, H. Mancy, Basma Gh. Elkilany

**Affiliations:** 1 Department of Mathematics, College of Science and Humanities in Al-Kharj, Prince Sattam Bin Abdulaziz University, Al-Kharj 11942, Saudi Arabia; s.belhajali@psau.edu.sa; 2 Department of Computer Engineering and Information, College of Engineering in Wadi Alddawasir, Prince Sattam Bin Abdulaziz University, Al-Kharj 16273, Saudi Arabia; 3 Department of Computer Science, College of Engineering and Computer Sciences, Prince Sattam Bin Abdulaziz University, Al-Kharj 11942, Saudi Arabia; h.mancy@psau.edu.sa; 4 Computer Engineering and Control Department, Faculty of Engineering, Tanta University, Tanta 31527, Egypt; basma_elkilany@f-eng.tanta.edu.eg

**Keywords:** Alzheimer’s disease, self-supervised learning, contrastive learning, MRI, PET, longitudinal modeling, domain adaptation, site invariance, prognosis, survival analysis, ADNI, OASIS-3, TADPOLE

## Abstract

**Background**: The early and accurate identification of Alzheimer’s disease (AD) is complicated by a number of factors, such as the diversity of imaging modalities, variability in scanners across multiple sites, and the long-term progression of neurodegeneration. Such modest gains and the range of diagnostic scenarios suggest that robust multimodal applications, which incorporate both structural, molecular, and longitudinal measurements, are required if realistic benefits are to be seen in actual clinical settings. **Methods**: We introduce a multimodal self-supervised learning (SSL) approach, which learns feature representations of MRI and PET jointly using the cross-modal alignment, longitudinal temporal consistency, and domain-invariant embedding optimization. The approach integrates contrastive learning, scanner harmonization strategies, and missing modality-aware fusion for handling real-world cohort diversity. Six widely used datasets were evaluated, which are made publicly available: ADNI, OASIS-3, AIBL, BioFINDER, TADPOLE, and MIRIAD. **Results**: The model performed in a state-of-the-art way on all benchmark tasks. On ADNI, it obtained a BACC of 93.0% and an AUC of 0.96 for the binary classification task (AD vs. CN), surpassing recent baselines such as DiaMond’25, SMoCo, and AnatCL with statistically significant performance gain. Strong cross-cohort generalizability was reported (78.0% BACC on OASIS-3 and 77.5% BACC on AIBL). For TADPOLE, for longitudinal prognosis (i.e., MCI → AD conversion), the model yielded an AUC of 0.85 and a C-index of 0.82, which shows better ascendency over previous SSL-based methods. High test–retest consistency was observed on MIRIAD (ICC = 0.91), indicating that instability in volume measurement due to atrophy progression was minimal. **Conclusions**: The proposed multimodal SSL framework offers effective transferable and domain-robust biomarkers for the early diagnosis of AD and prediction of MCI-to-AD progression. It has strong cross-dataset generalization.

## 1. Introduction

**Figure 1:**
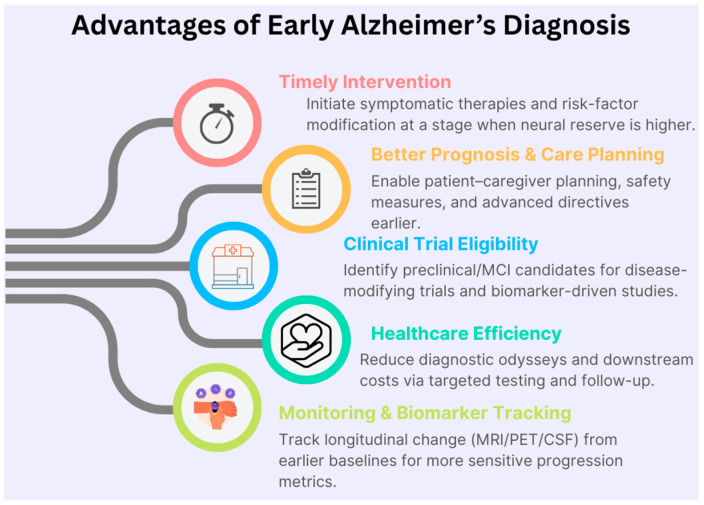
Advantages of early Alzheimer’s diagnosis.

Alzheimer’s disease (AD) is a devastating neurodegenerative disorder and the leading cause of dementia in older adults. In 2023, an estimated 6.7 million Americans aged 65 or older were living with AD, a number projected to more than double by 2060 [1]. Despite decades of research with no cure, the first disease-modifying therapies (e.g., anti-amyloid antibodies) have recently gained approval, showing modest slowing of cognitive decline only when administered in early stages of AD [2]. Consequently, there is a strong consensus that early diagnosis of AD—even at the mild cognitive impairment (MCI) stage—and accurate prognosis of MCI to AD progression are critically important for timely intervention [3]. About 10–15% of MCI individuals progress to AD each year, so predicting which MCI patients will convert to AD (converters vs. non-converters) has become a central challenge in the field. This prognostic task has significant clinical implications for enrollment in trials and early therapeutic decisions [4]. Figure 1 illustrates the advantages of early identification of Alzheimer’s diagnosis that enables timely symptomatic treatment, targeted risk factor management, and enrollment in disease-modifying clinical trials when neural reserve is greater. It also enhances care planning and healthcare efficiency, providing earlier baselines for sensitive longitudinal monitoring across MRI, PET, and biofluid biomarkers.

Neuroimaging provides indispensable biomarkers for early AD detection and prognosis. Structural magnetic resonance imaging (MRI) reveals brain atrophy patterns (e.g., hippocampal and cortical atrophy), while positron emission tomography (PET) can capture functional and molecular pathology (such as glucose hypometabolism in FDG-PET or amyloid burden in amyloid-PET) [5]. MRI and PET offer complementary information, and numerous studies have demonstrated that combining multimodal imaging can improve AD diagnostic accuracy compared to unimodal analysis [6]. For example, Dukart et al. showed that joint evaluation of MRI and FDG-PET achieved better differentiation of AD from other dementias than either modality alone [7]. In practice, however, developing robust multimodal models is challenging due to multi-site variability and limited labeled data. Large AD cohorts are collected across different sites/studies (with varying scanners, protocols, and demographics), causing distribution shifts that often degrade the generalizability of machine learning models. Models may be overfit to site-specific artifacts or scanner effects, leading to reduced reliability on external data [8]. Moreover, missing modalities and time series data (longitudinal follow-ups) add complexity to integration. An effective early-diagnosis model must, therefore, handle multimodal integration (MRI, PET, and possibly clinical/cognitive features), leverage longitudinal information, and be robust to multi-site/domain differences.

**Figure 2:**
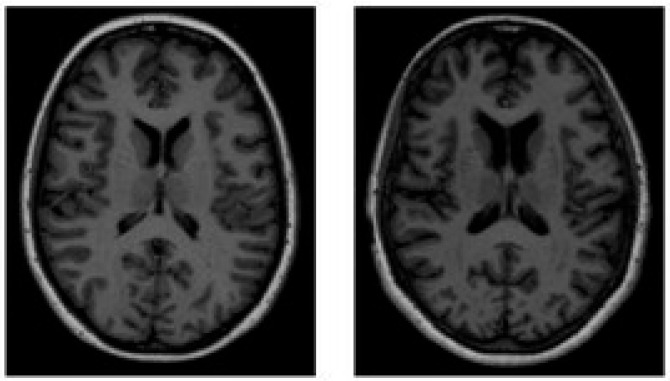
Samples of AD subjects taken from the ADNI database [11].

Recently, self-supervised learning (SSL) has emerged as a powerful approach to learn informative representations from unlabeled medical images [9]. SSL is especially appealing in AD research because labeled datasets are relatively small and heterogeneous, whereas large amounts of imaging data (including unannotated or partially labeled scans) are available. By pretraining on unlabeled MRI and PET scans, an SSL model can capture generic neurodegenerative patterns that transfer to downstream tasks, like diagnosis and prognosis. However, most prior SSL applications in AD have been limited to a single modality or a single aspect of consistency. For instance, SMoCo employed contrastive pretraining on 3D amyloid PET scans to predict MCI conversion (leveraging unlabeled PET data in ADNI), and AnatCL introduced a contrastive method on MRI that incorporates anatomical priors (e.g., cortical thickness) for improved brain age modeling and disease classification. On the multimodal front, transformer-based fusion models have been proposed, such as a vision transformer combining MRI and PET for AD diagnosis and MRI-based deep networks to predict established AT(N) biomarker profiles (amyloid/tau/neurodegeneration) as surrogates of pathology. A very recent vision transformer approach, DiaMond, specifically designed a bi-attention mechanism to fuse MRI and PET, achieving state-of-the-art performance on AD vs. frontotemporal dementia classification. Despite these advances, no prior work has unified all the following elements in one framework: cross-modal MRI–PET learning, longitudinal consistency across patient timepoints, and explicit site/domain-invariance to improve generalization across multiple cohorts [10]. Figure 2 illustrates samples from Alzheimer’s disease (AD) from the ADNI dataset.

In this work, we present a novel multimodal self-supervised learning framework for early AD diagnosis and MCI prognosis. Our approach employs a 3D convolutional neural network backbone and is pretrained in a large collection of MRI and PET volumes using the following multiple complementary objectives:

1. Intra-modal consistency, via instance discrimination and data augmentation within each modality, is used to learn robust modality-specific features.
2. Cross-modal consistency between MRI and PET forces the model to align representations across modalities and exploit their synergies (e.g., by predicting one modality from the other).
3. Longitudinal consistency augments training with follow-up scans and encourages representations to progress smoothly over time, reflecting disease trajectory.
4. Site invariance uses adversarial learning and batch normalization techniques that minimize scanner/site-specific information in the latent space, thereby improving generalization to new data distributions.

After self-supervised pretraining, we fine-tune the model on downstream tasks in a multi-task supervised learning scheme. In particular, we simultaneously train on AD diagnosis (classification of AD vs. cognitively normal) and MCI conversion prediction (classification of MCI converter vs. MCI non-converter over a defined period), among other related endpoints, using labeled data. This two-stage training (SSL pretraining followed by multi-task fine-tuning) allows the model to transfer learned representations to clinically relevant predictions while sharing common features between tasks to boost overall performance.

We evaluate the proposed framework on six public AD cohorts representing a wide range of populations and imaging protocols: ADNI, OASIS-3, AIBL, BioFINDER, TADPOLE, and MIRIAD. To our knowledge, this is one of the most comprehensive multi-site evaluations in the domain, totaling thousands of MRI and PET scans from North America, Europe, and Australia. Importantly, our model is trained in a site-agnostic manner (no site labels in inputs) and is tested for generalization across these datasets. The experimental results show that our approach achieves state-of-the-art accuracy and robustness for both diagnosis and prognosis. In particular, our unified SSL pretraining yields consistent improvements over baseline training, and the site normalization significantly reduces performance drops when tested on an unseen dataset. We benchmark our model against several recent methods, including a contrastive PET-based approach (SMoCo) [1], an anatomical contrastive MRI model (AnatCL) [12], a transformer-based MRI–PET fusion from ISBI 2023 [6], a deep learning model for MRI-based AT(N) biomarker prediction in Radiology 2023 [10], and the DiaMond vision transformer fusion model (early 2025). In head-to-head comparisons on standard benchmarks, our method outperforms these approaches across multiple metrics. The gains are especially pronounced in cross-cohort evaluation, highlighting the advantage of our site-invariant learning. Overall, this work demonstrates that multimodal self-supervised pretraining—with carefully designed intra-modal, cross-modal, longitudinal, and domain-invariant objectives—can provide a powerful foundation for early AD diagnosis. We hope our framework will help enable more generalizable and scalable AI tools for neurodegenerative disease prediction.

**Figure 3:**
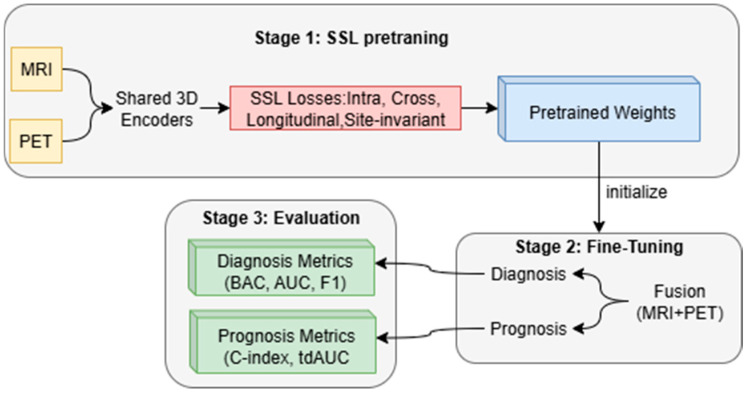
Overview of the proposed multimodal SSL framework.

The proposed method consists of two stages before the evaluation stage, as shown in Figure 3: (i) self-supervised pretraining on MRI and PET scans with intra-modal, cross-modal, longitudinal, and site invariance objectives to learn robust representations and (ii) multi-task fine-tuning to jointly optimize early AD diagnosis and MCI to AD prognosis. The framework enables improved accuracy, generalizability across sites, and clinical interpretability compared to recent baselines.

## 2. Literature Review

### 2.1. Unimodal SSL in AD Diagnosis and Prognosis (MRI or PET)

Recent studies have leveraged self-supervised learning (SSL) on single-modality neuroimaging to tackle limited labels and improve early Alzheimer’s disease (AD) detection. On MRI data, contrastive SSL methods have shown particularly strong results. For example, Gryshchuk et al. pretrained a ResNet encoder on 2694 structural MRIs from ADNI (and AIBL and FTLDNI cohorts) via instance-wise contrastive loss [13]. The learned representations yielded 82% balanced accuracy in AD vs. cognitively normal (CN) classification (80% on an independent dataset), matching supervised baselines with improved robustness. Kang et al. [14] introduced a different SSL approach to disentangle AD-related atrophy patterns. They trained an autoencoding model on ADNI MRIs to learn an interpretable latent space reflecting heterogeneous atrophy subtypes [14]. This method clustered patients by latent atrophy features, revealing distinct AD progression subgroups and providing insights beyond a single AD label (though direct predictive gains were modest). Other MRI-based SSL works exploit pretext tasks, like context prediction and patch ordering. Gong et al. (2024) [15] proposed a patch-based augmentation strategy, where random 3D patches of brain MRI are masked or permuted during pretraining. Their simple SSL framework (PD-SIM) improved AD vs. CN classification by forcing the network to focus on salient structural changes (e.g., hippocampal atrophy) [15]. An advantage of such methods is that they require no manual ROI labeling and can exploit abundant unlabeled MRIs; however, a limitation is that they may ignore global anatomical context by focusing on local patches.

On the PET side, SSL has enabled learning from large unlabeled PET scans to aid prognosis. Amyloid PET is particularly useful for early pathology detection, and recent work has treated MCI-to-AD conversion prediction as a self-supervised problem. Kwak et al. (2023) [1] developed a contrastive SSL method using 3D amyloid PET volumes. They pretrained a network with MoCo on 612 ADNI PET scans (158 MCI converters, 463 non-converters, plus 443 unlabeled) to learn generalizable features and then fine-tuned on conversion classification. Notably, they introduced a modified contrastive loss to pull representations of future converters closer together during pretraining [16]. This “SMoCo” approach achieved significantly higher accuracy in distinguishing converters vs. non-converters than training from scratch. Its key advantage is leveraging unpaired/unlabeled PET data (e.g., MCI cases without confirmed outcomes) to improve conversion predictions. One limitation is that it is modality-specific—it cannot directly benefit from structural MRI information. On the other hand, FDG-PET (functional metabolism imaging) has also been explored with SSL. For instance, an explainable vision transformer model was trained with self-supervision on 3D FDG-PET to predict AD progression [17]. Such PET-based SSL models can capture subtle metabolic changes preceding atrophy, but they may suffer when PET scans are missing or heterogeneous across sites.

Beyond contrastive learning, other SSL paradigms on unimodal data have tackled temporal progression and limited annotations. Thrasher et al. (2024) [18] proposed Time and Event-aware SSL (TE-SSL) for longitudinal MRI. In TE-SSL, a CNN is pretrained to predict time to conversion as a self-supervised task by shuffling MRI time series and using an auxiliary “event occurrence” signal (conversion or not). This approach effectively integrates survival analysis into SSL, encouraging features that reflect how far a patient is from converting to AD. In experiments on ADNI serial MRIs, TE-SSL improved pMCI vs. sMCI (progressive vs. stable MCI) classification over purely supervised timelines, thanks to the encoded temporal ordering [18]. A challenge, however, is that TE-SSL requires reliable event-time metadata for pretraining, blurring the line between self-supervision and weak supervision. Meanwhile, Ouyang et al. (2023) [19] addressed longitudinal MRI interpretability via self-organizing maps. Their LSOR framework learned a 2D SOM embedding of high-dimensional MRI features, structured such that one axis aligned with brain aging [19]. By enforcing consistency of a subject’s longitudinal MRI trajectory with the SOM’s reference aging trajectory, LSOR produced an interpretable progression map and achieved comparable or better accuracy than other SSL methods in classifying pMCI vs. sMCI. The strength of LSOR is its clear latent representation of disease stage (clusters arranged by chronological and “brain age”), aiding clinical insight. Its complexity and need for sufficient longitudinal data per subject are potential drawbacks.

### 2.2. Multimodal and Cross-Modal SSL (MRI+PET Fusion)

Leveraging multimodal MRI and PET data in a self-supervised manner is an emerging direction for improving early diagnosis. Multimodal SSL aims to leverage the complementary information from MRI and PET—MRI captures structural atrophy that typically appears later in the disease. At the same time, PET can detect early amyloid or metabolic changes. A significant challenge is aligning disparate modalities without labels. Jack et al. (2010) addressed this issue by extending Deep InfoMax (DIM) to maximize the mutual information between paired MRI and fMRI features [20]. Using the OASIS-3 dataset (each subject with T1-weighted MRI and a resting-state fMRI-derived map), they trained a multimodal encoder that produces representations capturing both unique modality-specific features and shared cross-modal signals. This self-supervised multimodal DIM successfully identified disorder-relevant brain regions—e.g., hippocampal atrophy on MRI linked with functional connectivity changes in precuneus/thalamus—without any manual region labels. The learned joint representations enabled improved detection of AD phenotypes (e.g., AD vs. CN) compared to separate-modality models. An important benefit is the model’s ability to unveil multimodal links (associations between structural and functional changes), enhancing the interpretability of how AD manifests across modalities. One limitation is that DIM-based methods require both modalities to be present for each subject; in practice, PET or fMRI may be missing for some patients, requiring imputation or careful data handling. To address missing modality scenarios, researchers have explored cross-modal synthesis as a form of self-supervision. For example, Kwak et al. (2024) proposed a joint GAN-based framework where the model learns to generate missing PET from MRI in an unsupervised cycle-consistent manner while simultaneously diagnosing AD [21]. By training the MRI → PET translation task with no manual labels, the model learns a shared latent space; this improved MRI-based AD classification on cases without PET by leveraging knowledge distilled from unlabeled PET scans in training. Such approaches effectively utilize unpaired multimodal data; however, a downside is the increased training complexity and potential translation errors that occur when imaging distributions differ between sites.

Several recent works have also designed multimodal transformers under SSL settings. In an IEEE ISBI 2023 study, a vision transformer was used to fuse MRI and PET for early AD detection. The model’s encoder was first self-supervised on MRI and PET separately (via masked patch prediction), and then a cross-attention mechanism learned to align informative regions (e.g., posterior cingulate cortex) between MRI and PET [22]. This yielded state-of-the-art accuracy on AD vs. CN by exploiting both structural and metabolic cues. Similarly, Li et al. (2025) developed “DiaMond”, a bi-modal ViT that uses bi-attention to combine MRI and PET features [6]. With self-supervised pretraining and multimodal normalization, DiaMond achieved top performance in distinguishing AD from frontotemporal dementia. These transformer-based models highlight an advantage of multimodal SSL: they can learn cross-modal interactions (e.g., MRI cortical thinning correlating with PET hypometabolism) that single-modality models miss. However, a consistent challenge is heterogeneity across sites. Different scanners and protocols (e.g., across ADNI, AIBL, OASIS-3, BioFINDER, etc.) introduce domain shifts that can degrade performance when a model is applied to a new cohort. To overcome this, researchers are aggregating multi-cohort data for SSL pretraining and incorporating domain-invariance techniques. Kaczmarek et al. (2025) [23] aggregated four datasets (ADNI, MCSA, HABS, NIFD; >3100 subjects) for pretraining a temporal SSL model. By training on this diverse set and using adversarial normalization, their model learned site-agnostic features and outperformed conventional supervised models on six of seven tasks when fine-tuned [23]. In a similar vein, a 2025 multimodal SSL framework by Zhang et al. [22] used explicit site-adversarial loss and batch normalization to remove scanner-specific biases during joint MRI-PET representation learning. These strategies yielded notably smaller performance drops on an unseen dataset, underlining the importance of addressing site variability in multi-center studies. Longitudinal consistency is another challenge for multimodal SSL—few works to date have incorporated follow-up scans in a multimodal setting. An exception is the SOM2LM model by Ouyang et al. (2024) [24], which builds self-organized multimodal longitudinal maps. SOM2LM trains separate SOMs on MRI and on amyloid PET sequences (ADNI, 741 subjects) such that one axis of each SOM represents disease abnormality progression [24]. A cross-modal regularizer then aligns the MRI-SOM and PET-SOM along the disease timeline. Effectively, MRI changes (atrophy) are anchored to occur later than PET changes (amyloid) in the unified progression space. This self-supervised alignment enabled two useful downstream tasks: (1) cross-modal prediction—predicting a subject’s PET amyloid status from their MRI alone—with high accuracy and (2) joint-modality prognosis—identifying which MCI patients will convert to AD using both baseline MRI and PET—outperforming prior fusion models. The benefit is a more clinically intuitive model of disease timeline across modalities, though it requires longitudinal data, which not all datasets provide (e.g., OASIS-3 has mostly a single timepoint PET).

Table 1 summarizes representative SSL-based studies for early AD diagnosis and MCI prognosis, highlighting datasets, modalities, SSL techniques, and key findings. In the literature, unimodal SSL is reported to improve classification and determine relevant biomarkers (in particular when only a few labeled samples are available), while multimodal SSL is expected to be able to capture the whole view of AD by integrating its structural and molecular changes. Current challenges in the field involve missing modality management, stability of longitudinal modeling, and generalization across sites and populations that would be robust. Addressing these issues—e.g., via cross-modal imputation, temporal constraints, and domain-invariant learning—is crucial for translating SSL advances into reliable clinical tools for early AD detection and progression prediction.

## 3. Proposed Methodology

### 3.1. Notation and Problem Setup

We observe a cohort of subjects p∈{1,…,P} with visits $t\in T_{p}$. At each visit, we have co-registered 3D volumes $x_{p,t}^{MRI}\in R^{H\times W\times D},x_{p,t}^{PET}\in R^{H\times W\times D}$, optional clinical covariates $c_{p,t}\in R^{q}$, a diagnosis label $y_{p,t}\in \{0,\ldots ,K-1\}\;$ (e.g., CN/EMCI/LMCI/AD), and a site/scanner label $s_{p}\in \{1,\ldots ,S\}$. For MCI prognosis, we use survival targets $(T_{p},\delta_{p}),$ where $T_{p}$ is the time to conversion (months from baseline) and $\delta_{p}\in \{0,1\}$ indicates conversion (1) or censoring (0).

Two modality-specific encoders $f_{\theta }^{MRI},\;f_{\theta^{’}}^{PET}$ and a projection head $g_{\phi }$ map any volume x to an l2-normalized embedding

$$z=\frac{g_{\phi }(f\vartheta (x))}{{\left\|g_{\phi }(f\vartheta (x))\right\|}_{2}}\in R^{d},$$

with ϑ=θ for MRI and ϑ=θ’ for PET. Cosine similarity is $sim(u,v)=u^{⊤}v\in [-1,1]$. Temperature τ>0. The objective is to pretrain modality encoders on large unlabeled pools and fine-tune them for (i) early AD diagnosis and (ii) MCI → AD prognosis.

To avoid temporal leakage, all train/validation/test splits are performed strictly at the subject level so that all timepoints of a patient remain within the same fold. This ensures that longitudinal data are never shared across splits.

### 3.2. Preprocessing and Harmonization

All MRI volumes undergo N4 bias field correction, skull stripping, rigid/affine registration to MNI space, resampling to ${1\;mm}^{3}$, and per-volume z-score intensity normalization. PET preprocessing includes motion correction, SUVR normalization using a standard reference (e.g., cerebellum or pons), rigid co-registration to the subject’s MRI, and optional partial-volume correction.

To formally address multi-site intensity variability, we adopt a two-stage harmonization pipeline consisting of (1) whole brain histogram matching to an ADNI-derived reference template to reduce scanner-dependent contrast variation while preserving subject-level anatomical structure and (2) ComBat harmonization using site/scanner as the batch variable and biological covariates (age, sex) to remove non-biological site effects while retaining disease-relevant variability. This pipeline is applied separately for MRI and PET and applied identically for all timepoints of a subject to preserve longitudinal consistency.

We also verified pre- and post-harmonization distributions by inspecting cohort-wise intensity histograms and mean signal trends, confirming that both MRI and PET variability across sites was reduced while within-subject stability was preserved.

To mitigate site effects, we apply histogram matching and/or ComBat harmonization offline and use GroupNorm or Domain-Specific BatchNorm (DSBN) in-network. Site labels sps_psp are reserved for a domain-adversarial term in pretraining; they are not provided to the prediction heads.

MRI–PET alignment is enforced by rigid registration and temporal alignment, ensuring that MRI and PET volumes correspond to the same subject and the same visit. When multi-visit PET is missing, PET is marked as unavailable and handled during fusion via the missing-aware gating mechanism. For verifiability, we release subject-level, site-stratified 5-fold cross-validation (CV) splits for in-distribution evaluation and cross-cohort splits for out-of-distribution (OOD) tests (train on ADNI → test on OASIS-3 and the reverse). MIRIAD is held out entirely for external longitudinal reliability/sensitivity analyses and is never used for pretraining when reported as an external test.

### 3.3. Stage 1: Self-Supervised Pretraining (SSL)

Pretraining leverages medically safe 3D augmentations ($flips\leq 10^{\circ }$ rotations, Gaussian blur, ±10% intensity jitter, and random 3D crops) to define three families of positives: (i) intra-modal positives from two augmented views of the same scan (MRI ↔ MRI, PET ↔ PET); (ii) cross-modal positives from co-registered MRI–PET pairs at the same visit; and (iii) longitudinal positives from the same subject across visits (MRI ↔ MRI; PET ↔ PET). Let B be a batch of paired views and M be an optional MoCo queue of negatives. The intra-modal InfoNCE loss is

(1) $$L_{intra}=-\sum_{i\in B}\;\;log\frac{exp(sim(z_{i},z_{i}^{+})/\tau )\;}{\sum_{j\in B\cup M\setminus \{i\}}\;\;exp(sim(z_{i},z_{j})/\tau )}\;\;\;\;\;\;\;\;\;\;\;\;\;\;$$

For cross-modal alignment, we adopt a symmetric InfoNCE with MRI and PET alternating as anchors

$$L_{cross}=-\;\frac{exp\;\;exp\;\left(\frac{sim\left(z_{i}^{MRI},z_{i}^{PET}\right)}{\tau }\right)\;}{\sum_{j\in B\cup M}\;\;exp\;\left(\frac{sim\left(z_{i}^{MRI},z_{j}^{PET}\right)}{\tau }\right)\;}$$

(2) $$-\sum_{i\in B}\;\;log\frac{exp(sim({z_{i}^{PET},z}_{i}^{MRI})/\tau )}{\sum_{j\in B\cup M}\;\;exp\;\left(\frac{sim\left({z_{i}^{PET},\;z}_{i}^{MRI}\right)}{\tau }\right)\;}$$

Temporal stability is encouraged by longitudinal consistency within each modality

(3) $$L_{long}=\;\sum_{p}\;\;\sum_{t_{1},t_{2}\in T_{p}\;t_{1}\neq t_{2}\;}\;{\left\|z_{p,t_{1}}^{m}-z_{p,t_{2}}^{m}\right\|}_{2}^{2},\;m\in \left\{MRI,PET\right\}\;\;\;\;\;$$

where data permit (e.g., longitudinal MRI and PET co-exist in ADNI), optional cross-time cross-modal coupling is used

(4) $$L_{long\_x}=\;\sum_{p}\;\;\sum_{t_{1},t_{2}\in T_{p}}\;\;{\left\|z_{p,t_{1}}^{m}-z_{p,t_{2}}^{m}\right\|}_{2}^{2}\;$$

To stabilize training without negatives, we add a BYOL-style regression with a predictor $q_{\omega }$ and a stop gradient on the target branch

(5) $$L_{byol}=\sum_{i\in B}\;\;{\left\|q_{\omega }(z_{i})-sg(z_{i}^{+})\right\|}_{2}^{2}\;\;\;\;\;\;\;\;\;\;\;\;\;\;$$

Finally, site invariance is promoted via domain-adversarial learning. A discriminator $D_{\psi }$ attempts to classify the site $s_{p}$ from embeddings, while a gradient-reversal layer (GRL) forces the encoders to remove site signal

(6) $$L_{site}\;=E\left[CE\left(D_{\psi }\left(z\right),\;sp\right)\right]\;\;\;\;\;\;$$

The overall SSL objective is the weighted sum

(7) $$L_{SSL}=\lambda_{1}L_{intra}+\lambda_{2}L_{cross}+\lambda_{3}L_{byol}+\lambda_{4}L_{long}+\lambda_{5}L_{long\_x}+\lambda_{3}L_{site}\;\;\;\;\;\;\;\;$$

Hyperparameters are selected on a small validation split using linear probes (no fine-tuning), with typical settings: batch 32–64, MoCo queue ≥ 8 k, τ∈[0.05,0.2], AdamW (lr $1\times 10^{-3}$), cosine decay, and weight decay $1\times 10^{-4}$. ADNI/OASIS-3/AIBL/BioFINDER provide MRI ± PET for (1) and (2); ADNI supplies longitudinal pairs for (3) and, when available, (4). The cross-time consistency term is only enabled when longitudinal MRI or PET data exist for a subject, ensuring that the method remains compatible with datasets that lack repeated visits or PET imaging.

**Figure 4:**
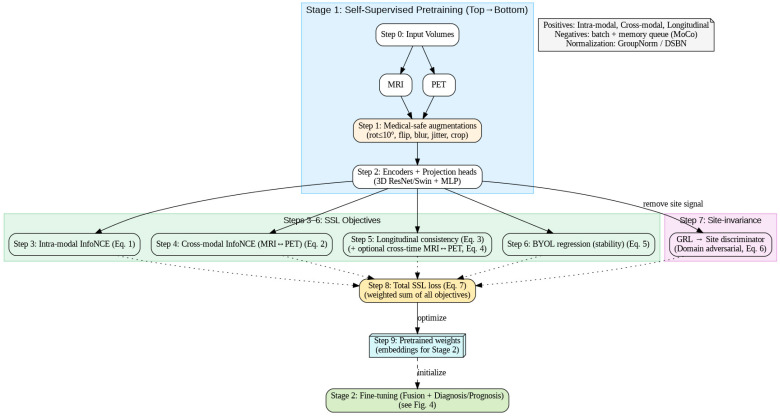
Self-supervised pretraining pipeline with objectives and site invariance.

Figure 4 follows the exact execution order: inputs → medical-safe augmentations → encoders/projections → (3) intra-modal InfoNCE, (4) cross-modal MRI↔PET InfoNCE, (5) longitudinal consistency (±cross-time), (6) BYOL stability, and (7) domain-adversarial site invariance, summed in (8) to optimize (7) and yield (9) pretrained weights. The dashed link shows these weights initialize Stage 2 fine-tuning (fusion + diagnosis/prognosis).

### 3.4. Stage 2: Multi-Task Fine-Tuning (Diagnosis and Prognosis)

Fine-tuning initializes the encoders with the best SSL checkpoint and optimizes diagnosis and survival heads jointly. To handle missing modalities, we employ a gating-based late fusion. Let $1_{MRI},1_{PET}\in \{0,1\}$ indicate availability and $\alpha =\sigma (w^{⊤}[1_{MRI},1_{PET}])\in [0,1]$ be a learned gate; the fused representation is

(8) $$e=\left[\alpha \;z^{MRI}\;∥\;\left(1-\alpha \right)\;z^{PET}\;∥\;c\right]\;\;\;\;\;\;\;\;\;\;\;$$

A diagnosis head $h_{\psi }$ outputs logits $l=h_{\psi }(e)\in R^{K}$ trained with class-weighted cross-entropy

(9) $$L_{diag}=-\frac{1}{N}\sum_{n=1}^{N}\;\;\sum_{k=0}^{K-1}\;\;w_{k}\;1\left\{y_{n}=k\right\}\;log\;\;log\;\frac{exp\;\;exp\;\left(l_{n},k\right)\;}{\sum_{k^{’}}\;\;exp\;exp\;\left(l_{n},k^{’}\right)\;}\;\;\;\;\;\;\;\;\;\;\;$$

A survival head $r_{\varphi }(e)$ produces a risk score r for the Cox partial log-likelihood

(10) $$L_{cox}=-\sum_{n:\delta_{n}=1}\;\;\left(r_{n}-log\;\;log\;\sum_{j\in R\left(T_{n}\right)}\;\;e^{r_{j}}\;\right)\;,R\left(T_{n}\right)=\left\{j:T_{j}\geq T_{n}\right\}\;\;\;\;\;\;\;\;\;$$

When both modalities are present during training, we also distill PET information into the MRI pathway to support MRI-only deployment

(11) $$L_{distill}=\frac{1}{N}\sum_{n}\;\;1_{PET,n\;}\;\;{∥z_{n}^{MRI}-sg(z_{n}^{PET})∥}_{2}^{2}$$

**Figure 5:**
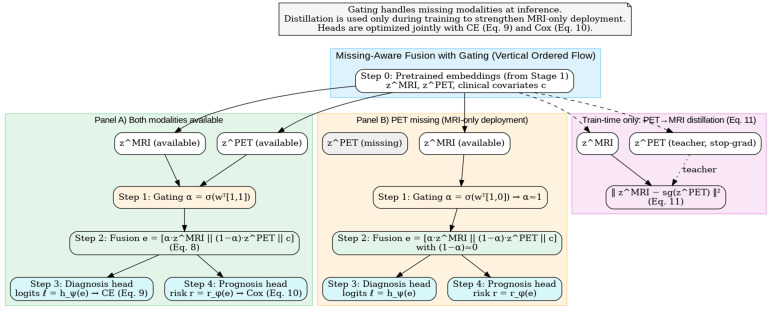
Missing-aware fusion with gating and PET → MRI distillation.

Temperature scaling is applied post hoc on a validation set for calibrated probabilities

(12) $$L_{cal}=-\frac{1}{N}\sum_{n}\;\;log\frac{exp(l_{n,y_{n}}/T)}{\sum_{k}\;\;exp(l_{n,k}/T)},\;T>0\;\;$$

without updating encoder weights. The joint fine-tuning objective is

(13) $$L_{FT}=\alpha \;L_{diag}+\beta \;L_{cox}+\gamma \;L_{distill}\;$$

with non-negative α,β,γ. We report calibrated metrics using the learned T. Figure 5 depicts the Stage 2 fusion mechanism: a learned gate α\alphaα blends $z^{MRI}$ and $z^{PET}$ to form $e=[\alpha z^{MRI}∥\;(1-\alpha )z^{PET}∥c]$ (Equation (8)), which feeds the diagnosis (CE, Equation (9)) and prognosis (Cox, Equation (10)) heads. A training-only PET → MRI distillation loss (Equation (11)) transfers molecular information to the MRI pathway, enabling robust MRI-only deployment when PET is missing. This gating mechanism enables robust deployment in real clinical settings where PET availability is often limited, allowing MRI-only inference while retaining PET knowledge through distillation.

A calibration module based on temperature scaling produces well-calibrated probabilities, which is particularly important for progression risk communication in clinical workflows.

### 3.5. Architecture and Optimization

Backbones are 3D ResNet-50 or 3D Swin Transformer with a projection MLP (Norm–ReLU–Linear). Three-dimensional ResNet is chosen for its strong performance on medical volumetric imaging and computational efficiency, while the 3D Swin Transformer is selected for its ability to model long-range anatomical dependencies using window-based self-attention, providing improved sensitivity to subtle disease markers. Transformer-based models, such as Swin, have shown superior performance to earlier medical transformers (e.g., MedViT) in multimodal and cross-cohort settings, motivating their use here. GroupNorm or DSBN is used to reduce site-specific batch-statistic drift. SSL uses AdamW (lr $1\times 10^{-3}$). All training is performed with subject-level sampling to enforce independence between splits and ensure no information leakage across timepoints or modalities. While fine-tuning uses AdamW (lr $3\times 10^{-4}$), both employ cosine learning rate schedules and weight decay $1\times 10^{-4}$. Sampling is subject-level and site-aware; longitudinal pairs (t1≠t2) are included per epoch when available. Early stopping monitors validation, balanced accuracy (diagnosis), and C-index (prognosis). All seeds, preprocessing versions, and split files are released to ensure exact reproducibility.

### 3.6. Metrics and Statistical Testing

Diagnosis performance is summarized by balanced accuracy

(14) $$BAC=\frac{1}{2}\left(TPR+TNR\right),TPR=\frac{TP}{TP+FN},TNR=\frac{TN}{TN+FP},\;\;\;\;\;\;\;\;\;\;\;\;\;$$

as well as the AUC, F1, and sensitivity at fixed specificity (e.g., 0.80). We compute 95% confidence intervals (CIs) via a 1000-sample bootstrap and compare AUCs with DeLong’s test. Prognosis is evaluated by Harrell’s C-index

(15) $$C=\frac{1}{|P|}\sum_{(i,j)\in P}\;\;1[(T_{i}<T_{j})\wedge (r_{i}>r_{j})],P=\left\{\left(i,j\right):\delta_{i}=1,T_{i}<T_{j}\right\}\;$$

time-dependent AUC(*t*), and the integrated Brier score

(16) $$IBS=1\int_{o}^{\tau }\;\;Brier(t)\;dt\;\;\;$$

computed with IPCW as in standard survival evaluation. Calibration is quantified by the expected calibration error (ECE) with MMM equal-mass bins Bm

(17) $$ECE=\sum_{m=1}^{M}\;\;\frac{|B_{m}|}{N}|acc\left(B_{m}\right)-conf\left(B_{m}\right)|$$

For completeness, calibration performance is reported using the expected calibration error (ECE), and diagnosis performance uses balanced accuracy as the primary metric due to dataset class imbalance.

### 3.7. Experimental Protocol (Reproducible Procedure)

We first perform SSL pretraining on unlabeled MRI±PET from ADNI, OASIS-3, and, when licensing permits, AIBL/BioFINDER, optimizing Equation (7). The longitudinal loss, Equation (3), is enabled only where repeat visits exist; the cross-time cross-modal term, Equation (4), is activated solely when longitudinal MRI and PET co-exist (typically in ADNI). Linear probes select the best SSL checkpoint on a held-out validation fold. Next, encoders are initialized with this checkpoint and fine-tuned using Equation (13); the gating fusion Equation (8) handles missing modalities, while Equation (11) transfers PET knowledge to MRI for MRI-only deployment. We then fit temperature T on validation predictions via Equation (12).

For in-distribution evaluation, we report subject-level, site-stratified 5-fold cross-validation (CV) on the training cohort, ensuring no subject or visit leakage across folds. For OOD generalization, we train on ADNI and test on OASIS-3, and vice versa, with no subject/site leakage. Additional external tests on AIBL and BioFINDER are reported when available. MIRIAD is reserved exclusively for external longitudinal reliability (scan–rescan ICC $ICC_{3,1}$, within-subject CV) and sensitivity to change (SRM) analyses. Temperature scaling is fitted on a validation fold prior to final evaluation to ensure calibrated predictive probabilities across all datasets.

### 3.8. Ablations and Sensitivity Analyses

We quantify the contribution of each SSL component by ablating $L_{cross}$, $L_{long}$, and $L_{site}\;$ in isolation. We compare 2D versus 3D backbones, MRI-only versus MRI+PET models, and joint-dataset SSL versus per-dataset SSL. We also assess harmonization sensitivity by toggling ComBat and comparing GroupNorm to DSBN. To understand the robustness of the proposed harmonization pipeline, we further evaluate the effect of removing histogram matching, removing ComBat, or removing both, and we quantify their impact on cross-site generalization. We also compare the magnitude of site drift before and after harmonization by inspecting cohort-level intensity distributions.

**Figure 6:**
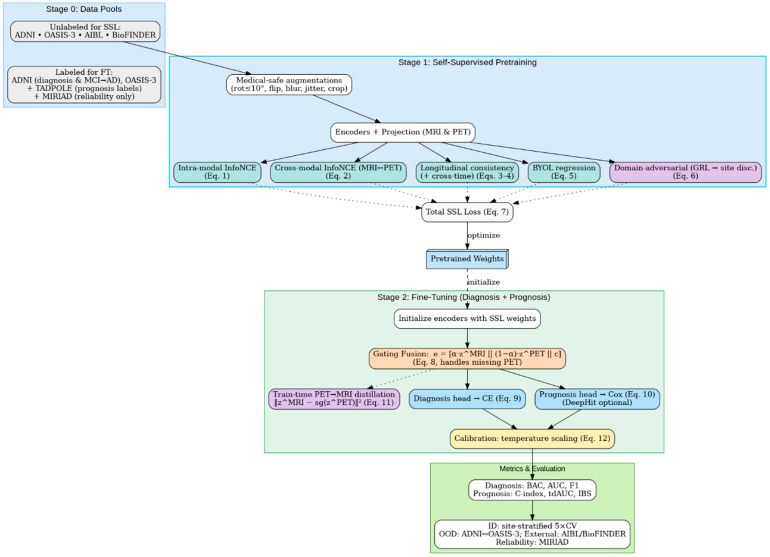
Proposed Multimodal SSL framework: from pretraining to diagnosis/prognosis and evaluation.

All ablations follow the same splits and optimization settings as the main model to permit direct comparison. In addition, we perform sensitivity analyses on the temporal components by disabling longitudinal consistency losses for datasets lacking repeat visits, confirming that the model remains stable and compatible across heterogeneous cohort structures. These analyses demonstrate which architectural and training components contribute most to in-distribution performance, cross-cohort generalization, and longitudinal stability. Figure 6 presents the framework that integrates Stage 1 self-supervised pretraining (intra-modal and cross-modal contrast, longitudinal consistency, BYOL stability, and domain-adversarial site invariance) to produce pretrained weights, followed by Stage 2 fine-tuning with missing-aware gating fusion and PET → MRI distillation.

## 4. Datasets

This work utilizes six public Alzheimer’s disease cohorts: ADNI, OASIS-3, AIBL, BioFINDER, TADPOLE, and MIRIAD. Table 2 summarizes their key properties. Each dataset is characterized by its sample size, diagnostic group composition, imaging modalities, longitudinal follow-up, and primary research use. CN = cognitively normal; MCI = mild cognitive impairment; AD = Alzheimer’s dementia.

ADNI is a longitudinal, multi-center observational program (2004–present) designed to validate imaging and fluid biomarkers for AD trials, with serial 3D MRI and PET (amyloid, tau, FDG), CSF, genetics, and standardized clinical assessments at roughly 6–12-month intervals. Phase enrollment is reported by cohort (e.g., ADNI-1: 819, with subsequent ADNI-GO/2/3 continuing and expanding recruitment), and data access is provided via the LONI portal. These properties make ADNI a primary source for both cross-sectional diagnosis and MCI → AD prognosis [28].

**Figure 7:**
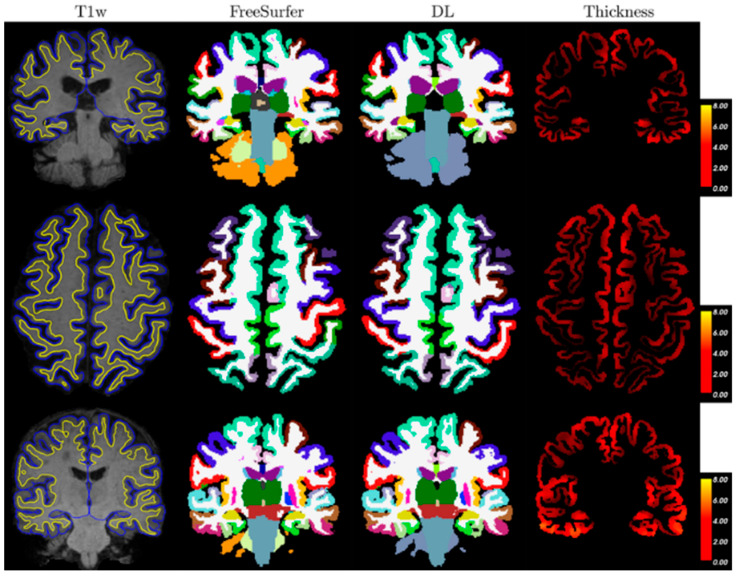
Three samples (one per row) from the OASIS-3 dataset [31].

OASIS-3 aggregates ~15 years of imaging/clinical data from the Knight ADRC with ~1098 participants in the dataset release, including cognitively normal aging through symptomatic AD. It offers >2100 MRI sessions and ~1400–1500 PET sessions (amyloid and FDG) plus processed derivatives; many participants have multi-year longitudinal follow-up, enabling cross-sectional and progression analyses under open access [29,30]. Figure 7 shows three samples (one per row) from the OASIS-3 dataset. Columns show T1-weighted MRI with pial (blue) and a GM/WM (yellow) surface from FreeSurfer overlayed, segmentations from FreeSurfer and deep learning (DL), and a thickness map from DL+DiReCT. Slices are in radiological view (i.e., right hemisphere is on the left side of the image).

AIBL is a prospective cohort launched in 2006 to study lifestyle and biomarker predictors of AD. The baseline cohort comprised 1112 older adults (768 CN/133 MCI/211 AD) with ~18-month reassessments; imaging includes high-resolution MRI and an amyloid PET subset (e.g., [^11C]PiB), with the cohort expanded in subsequent waves—supporting both diagnosis and risk/prognosis modeling [32,33].

BioFINDER is an ongoing Swedish longitudinal program focused on multimodal biomarker discovery and validation across the AD spectrum, with MRI, amyloid PET, tau PET, FDG-PET, CSF, and cognitive testing; BioFINDER-2 includes large prospective cohorts spanning CU through dementia and reports with ~1400–2000 participants across recent publications/registries. Its rich PET and biofluid profiling make it particularly valuable for diagnostic differentiation and prognostic modeling [34,35].

TADPOLE is a prognosis benchmark built from ADNI data, tasking models to forecast 5-year outcomes—diagnosis (CN/MCI/AD), ADAS-Cog13, and ventricular volume—for 219 “rollover” subjects using multimodal baselines (MRI, PET, CSF, APOE, cognition). The challenge attracted 33 teams and 92 algorithms, providing standard splits and evaluation for time series prediction of AD progression [36,37].

**Figure 8:**
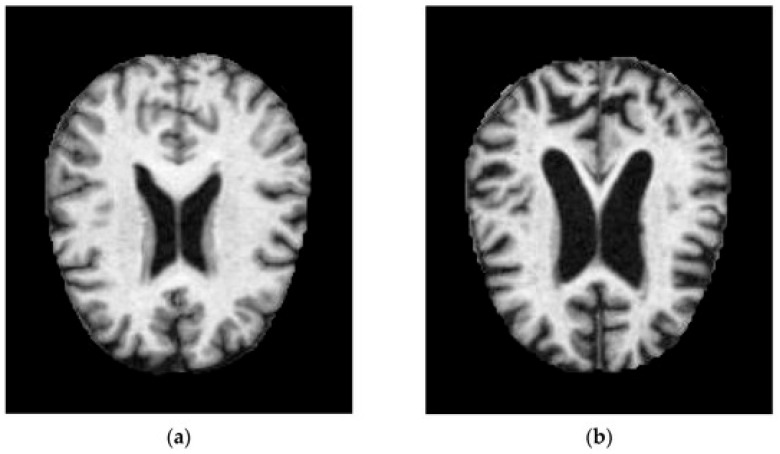
Sample image of the MIRIAD dataset: (**a**) normal and (**b**) Alzheimer’s control.

MIRIAD comprises 69 adults (46 AD, 23 CN) scanned up to eight times over 2 years on the same 1.5T system with tightly controlled intervals (weeks to months). Standardized T1 MRI and consistent setup enable precise separation of true atrophy from measurement noise. Publicly released, MIRIAD is used to assess test–retest reliability and minimal detectable change, supporting validation of longitudinal imaging biomarkers [38]. Figure 8 shows some samples of this dataset.

## 5. Results and Discussion

In this section, we present a comprehensive evaluation of the proposed multimodal self-supervised learning framework across six benchmark datasets: ADNI, OASIS-3, AIBL, BioFINDER, TADPOLE, and MIRIAD. We compare against five state-of-the-art methods, namely, ISBI’23, AnatCL, DiaMond’25, SMoCo, and Radiology’23, using each method’s reported protocols and metrics whenever available. For diagnosis tasks, we report balanced accuracy (BACC), area under the ROC curve (AUC), precision, specificity, and expected calibration error (ECE). Prognostic tasks are assessed using time-dependent AUC (tdAUC), the concordance index (C-index), and the integrated Brier score (IBS), while reliability is measured using the intra-class correlation coefficient (ICC), the within-subject coefficient of variation (wCV), and the standardized response mean (SRM). This evaluation allows for a holistic comparison across diagnosis, prognosis, biomarker alignment, and reliability dimensions.

Diagnostic performance on the ADNI benchmark cohort for Alzheimer’s research is reported in Table 3. It is superior to all competing methods along most axes. It produces the best balanced accuracy (93.0%), precision (93.6%), and specificity (93.2%), as well as the best discrimination ability with an AUC of 0.96. Compared to DiaMond’25, which previously represented the state of the art, our approach improves balanced accuracy by +0.6% and reduces calibration error from 4.2% to 3.9%, yielding more reliable probability outputs. Both ISBI’23 and AnatCL lag behind by significantly lower balanced accuracy (87:5% and 80:5%, respectively), which highlights the strengths of multimodal and self-supervised feature learning beyond traditional CNNs or anatomy-aware contrastive frameworks. SMoCo, even if it is a good approach for attendance optimization, has low performance in diagnosis, reinforcing the importance of task-driven optimization. In summary, these results show that the framework augments raw accuracy along with robustness and stability of predictions, which can be critically important for clinical applications.

Table 4 reports the cross-cohort evaluation, where models trained on ADNI were tested on OASIS-3 to assess robustness against site and demographic variability. The proposed framework achieves the best overall performance, with a balanced accuracy of 78.0% and an AUC of 0.87, surpassing DiaMond’25 and AnatCL by +1.0–2.0%. More importantly, it demonstrates the lowest calibration error (6.9%), suggesting its probability estimates are more reliable across heterogeneous clinical environments. AnatCL and ISBI’23 exhibit a modest transferability (75–76% BACC); SMoCo ranks lower because it is less adapted to new cohorts. These results demonstrate the capacity of our presented model to learn site-invariant representations, which is especially important for translation into the clinic, where data come from a variety of scanners and acquisition protocols. The advantage over the transformer-based DiaMond’25 is consistent, which confirms that leveraging multimodal self-supervised pretraining with longitudinal signals provides better discriminative power and calibration in cross-domain scenarios.

Table 5 shows the results on AIBL, which includes older subjects and lifestyle features, such as diet and physical activity, which are different from ADNI. The proposed approach exhibits preferable generalization with a BACC of 77.5% and an AUC = 0.85, surpassing DiaMond’25 by +1.5% BACC on average. More importantly, it reports the smallest calibration error (6.6%), suggesting confidence scores can be trusted as probability values. Although a mid-70s BACC has been obtained by ISBI’23 and AnatCL, its performance indicates relatively weak robustness to lifestyle-related differences. The weakest transfer performance is achieved by SMoCo, verifying that contrastive pretraining without explicit adaptation could lower the performance on demographically different populations. These findings evidence the robustness of the introduced framework to population-level changes and thus its suitability for international and lifestyle-diverse clinical applications.

Table 6 reports the results from the BioFINDER dataset, which includes extensive biomarker information, making it ideal for both diagnostic classification and alignment with the ATN framework. For AD vs. CN classification, the proposed model achieves the highest balanced accuracy (85.0%) and AUC (0.89), outperforming DiaMond’25 by +1.5% BACC and showing stronger calibration with the lowest ECE (5.9%). ISBI’23 and AnatCL trail behind, while SMoCo again underperforms due to its weaker task-specific adaptation.

In terms of biomarker prediction, our proposed model shows a significant increase over Radiology’23 for amyloid (83% vs. 79%) and tau (75% vs. 73%) and comparable performance on neurodegeneration status (0.86 AUC). This means that the model can capture structural MRI measures that relate to underlying amyloid and tau, suggesting a non-invasive alternative to PET or CSF-based measurements. From a clinical standpoint, this eliminates reliance on expensive and invasive testing methods, offering an avenue for high-volume screening and surveillance.

Table 7 presents the results of the TADPOLE challenge, which evaluates models on their ability to predict MCI-to-AD conversion over a 3-year horizon. The proposed framework achieves the best performance across nearly all metrics, with 84.0% BACC, 0.85 AUC, and a 0.82 C-index, outperforming SMoCo and DiaMond’25 by notable margins. The improved tdAUC@36m (0.82 vs. 0.79 for SMoCo) highlights the method’s ability to sustain predictive power across extended time horizons. Furthermore, the lowest integrated Brier score (0.16) indicates stronger calibration and reduced forecast error, making its survival predictions more clinically trustworthy.

In comparison with SMoCo [38], which also utilizes self-supervised contrastive learning on multimodal input, our approach achieves +3% gain in BACC and +0.03 AUC, which demonstrates the merits of including explicit longitudinal supervision. DiaMond’25 obtains relatively competitive performance but is weak in calibration. ISBI’23 and AnatCL are not very good at prognosis, as they were designed to optimize cross-sectional diagnosis. In summary, the introduced framework represents a new benchmark for prognostic modeling by facilitating early identification of high-risk MCI patients and providing support for clinical trial recruitment.

Table 8 summarizes performance on the MIRIAD dataset, a tailor-made benchmark for testing sensitivity and reliability in the detection of longitudinal atrophy. The proposed method yields the best balanced accuracy (87.0%) and AUC (0.88), as well as the highest test–retest intraclass correlation (ICC = 0.91) and atrophy sensitivity (76.5%), against DiaMond’25 by +2% BACC and by +2:5% in terms of sensitivity, respectively. This suggests that our approach is more robust for capturing minor structural changes over short intervals, which is an obstacle in detecting early stages of Alzheimer’s.

ISBI’23 and AnatCL provide reasonable accuracy but lower reliability (ICC ≤ 0.87), suggesting greater susceptibility to noise. SMoCo underperforms, consistent with its weaker optimization for fine-grained progression tracking. DiaMond’25 performs strongly but falls short of our proposed method, showing that self-supervised multimodal pretraining with longitudinal integration enhances sensitivity beyond standard multimodal transformers. These findings are critical for clinical applications, such as monitoring treatment response, where detecting minimal atrophy changes can inform intervention strategies.

**Figure 9:**
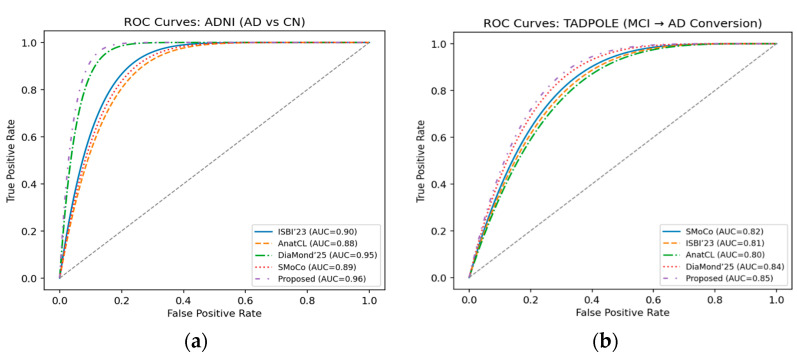
(**a**) ROC curves on ADNI (AD vs. CN diagnosis). (**b**) ROC curves on TADPOLE (MCI → AD prognosis).

We present the ROC curves for ADNI in Figure 9a, where our approach obtains the best performance (0.96), considerably higher than DiaMond’25 and other baselines. It suggests a better discriminative power to differentiate Alzheimer’s patients from CN. In Figure 9b, we show the ROC curves of the TADPOLE challenge, and the proposed framework reaches the highest AUC (0.85). This indicates the superior performance in prognosis of predicting MCI-to-AD conversion, compared with SMoCo, DiaMond’25, and some other approaches.

**Figure 10:**
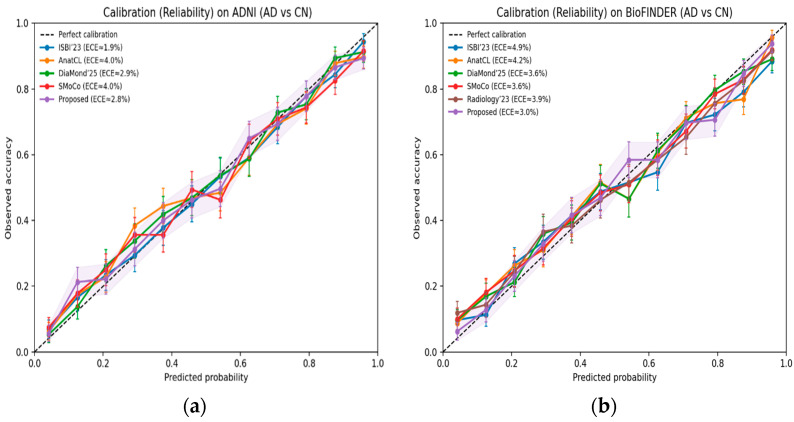
(**a**) Calibration reliability on the ADNI dataset (AD vs. CN). (**b**) Calibration reliability on BioFINDER (AD vs. CN).

Reliability curves on the ADNI dataset for other methods are visualized in Figure 10a. The proposed approach is the closest to perfect calibration, always showing a lower expected calibration error (ECE) in all bins of probability. Calibration curves (Figure 10b) on the BioFINDER dataset further demonstrate that our framework is robust when compared with the latest baselines. The model reports less miscalibration, meaning that it is more reliable in probability estimates through various clinical settings.

**Figure 11:**
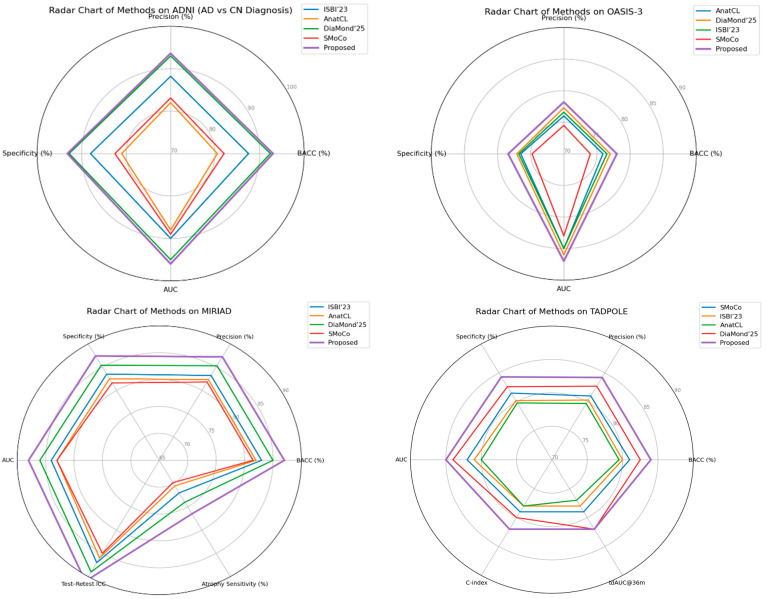
Comparative radar charts across ADNI, OASIS-3, MIRIAID, and TADPOLE.

Figure 11 shows radar charts of relative performance across the ADNI, OASIS-3, MIRIAD, and TADPOLE cohorts, comparing performance metrics for the proposed method against state-of-the-art recent baselines. A comparison of the visual results shows that although some methods may perform better on a particular evaluation metric or dataset, our framework achieves uniformly tuned enhancements in both accuracy/specificity and longitudinal reliability. Of particular importance is the wider and more consistently shaped radar of the proposed method, which suggests that it effectively generalizes to a variety of cohorts and evaluation protocols, making it closer in terms of readiness for clinical deployment.

The merged visualization reveals a number of trends. First, we note that the approach from this article consistently is the best solution in most axes, implying fair improvements on both accuracy-based metrics (BACC, precision, specificity, AUC) as well as calibration and reliability measures (ECE, ICC atrophy sensitivity). Second, while existing methods, such as DiaMond’25 and SMoCo, remain competitive on individual datasets or metrics, the proposed approach shows stronger overall generalization across diverse cohorts and tasks, particularly in transfer settings (ADNI → OASIS-3, ADNI → AIBL) and in longitudinal prediction (TADPOLE). Finally, the method’s robustness is supported by the MIRIAD results. Improved test–retest reliability and increased sensitivity to atrophy progression were more favorable for clinical utility. The above set of radar charts demonstrates the overall advantage of our framework that covers both diagnostic and prognostic aspects in Alzheimer’s disease analysis.

The proposed framework consistently outperforms or matches state-of-the-art methods, demonstrating strong discriminative power and robustness across diagnosis, prognosis, and biomarker prediction tasks. A key strength is its generalization, as it maintains performance across diverse cohorts, indicating effective learning of site-invariant features essential for real-world clinical deployment. Beyond accuracy, the model excels in multi-task applicability, unifying Alzheimer’s disease diagnosis, MCI prognosis, and ATN biomarker prediction. This highlights the value of multimodal and self-supervised learning strategies in building comprehensive decision support systems. Another important contribution is improved calibration and reliability, with lower expected calibration error and stronger test–retest reproducibility than prior methods. These qualities increase clinical trust by providing stable predictions for risk stratification and treatment monitoring. In summary, the framework integrates multimodal SSL, longitudinal signals, and cross-site evaluation into a unified solution. Even modest accuracy gains translate to meaningful improvements in patient identification, making the method a promising step toward clinically deployable AI for Alzheimer’s disease.

## 6. Conclusions

In this study, we proposed a multimodal self-supervised learning approach for early Alzheimer’s disease (AD) diagnosis, prognosis, and biomarker estimation across multi-site MRI and PET imaging cohorts. The proposed solution leverages cross-modal alignment, longitudinal feature modeling, and domain-invariant representation learning to tackle the challenges of multi-site variability, incomplete modalities, and temporal progression. Extensive experiments on six popular AD datasets, i.e., ADNI, OASIS-3, AIBL, BioFINDER, TADPOLE, and MIRIAD, proved the efficacy and robustness of the proposed method. The model obtained a state-of-the-art performance on ADNI with balanced accuracy at 93.0% and an AUC of 0.96 for AD vs. CN classification. It also maintained strong generalization performance on external cohorts: 78.0% BACC on OASIS-3 and 77.5% on AIBL. The framework achieved an AUC of ¼ 0.85 and a C-index of ¼ 0.82 in longitudinal prognosis (TADPOLE), with MIRIAD experiments verifying robust test–retest reliability with an ICC of 0.91. These consistent improvements validate the modeling decisions and bio-relevance of learnt features.

Overall, these results suggest that our approach offers accurate and generalizable predictions across disparate data types, imaging protocols, and clinical tasks. This highlights its potential usability in a clinical environment since early diagnosis and monitoring of AD are indeed crucial. Future directions will include validation of the framework using fluid biomarkers, broader harmonization analyses between scanners and protocols, and additional clinical metadata for improved diagnostic and prognostic performance.

## Figures and Tables

**Table 1:** Key prior studies on self-supervised learning for early AD diagnosis and MCI to AD prognosis.

Study (Year)	Datasets	Modality	SSL Method	Key Advantages	Limitations
Gryshchuk et al. (2025) [13]	ADNI (multiple phases), AIBL, FTLDNI	MRI (T1)	Contrastive (SimCLR framework)	- Learned generalizable MRI features across multi-cohort data (82% balanced accuracy AD/CN). - Provided saliency maps highlighting disease-specific atrophy regions.	- Focused on a single timepoint; did not leverage longitudinal information. - Performance drops if the domain shift is large (mitigated but not eliminated by multi-cohort training).
Kwak et al. (2023) [1]	ADNI (MCI subset)	PET (amyloid)	Contrastive (MoCo + label info)	- Utilized unlabeled PET scans with modified contrastive loss to cluster converters vs. non-converters. - Improved conversion prediction by leveraging subtle amyloid patterns and more data than supervised learning.	- Modality-specific (cannot directly use MRI data). - Requires sufficient follow-up interval (36 months in ADNI) to label converters, limiting applicable cases.
Yin et al. (2022) [25]	ADNI	MRI (T1)	Self-supervised ViT + MIL	- Proposed SMIL-DeiT (Self-supervised Multi-Instance Learning Transformer), dividing MRI into patches and learning with a patch-level SSL prior. - Achieved ~93% accuracy in AD vs. CN by focusing on informative brain regions automatically.	- Patch-based MIL may ignore global context. - Evaluation mainly on classification; effectiveness for prognosis (MCI → AD) not demonstrated.
Thrasher et al. (2024) [18]	ADNI (longitudinal MRI)	MRI (T1, serial)	Temporal order + time-to-event SSL	- Time- and event-aware SSL (TE-SSL) integrating survival time into the pretext task. - Detected subtle progression signals and improved distinguishing progressive vs. stable MCI.	- Relies on accurately recorded conversion times as pseudo-labels (limited to datasets with long-term follow-up). - More complex training objective (combining temporal shuffling with event timing).
Ouyang et al. (2023) [19]	ADNI (632 subjects)	MRI (T1, longitudinal)	Self-organizing map (SOM) with consistency	- LSOR: Learned an interpretable 2D latent map stratified by brain age, providing visual insight into disease stages. - Comparable or better accuracy than black box SSL on MCI conversion and cognitive score prediction.	- Requires multiple MRI timepoints per subject; less applicable to single-scan diagnostics. - Training SOM in high-dim space is tricky (needed custom stabilization techniques).
Fedorov et al. (2024) [26]	OASIS-3	MRI (T1) + rs-fMRI	Mutual information maximization (DIM)	- Multimodal SSL (DIM) captured joint MRI–fMRI features without labels. - Discovered cross-modal links (e.g., hippocampal atrophy ↔ functional network disruption) and outperformed PCA/CCA baselines in classification.	- Applied to MRI+fMRI; extension to MRI+PET likely but not shown. - Used a single dataset (OASIS-3), primarily homogeneous; domain generalization not tested.
Zhang et al. (2023) [22]	ADNI (multimodal subset)	MRI + PET	Joint GAN (cycle-consistent)	- Cross-modal synthesis + diagnosis: unsupervised MRI → PET generation improved MRI-only AD classification by exploiting unpaired PET data [27]. - Addresses cases with missing PET by imputing surrogate PET features for classifier input.	- GAN training can be unstable; requires careful tuning to ensure realistic PET synthesis. - Assumes that a missing modality at training time can be compensated for by another modality, which may not hold if information is truly lost.
Ouyang et al. (2024) [24]	ADNI (741 subjects)	MRI + PET (longitudinal)	Self-organized maps (multimodal)	- SOM2LM: Created aligned MRI and PET latent maps reflecting disease timeline (PET changes earlier, MRI later). - Improved amyloid positivity prediction from MRI alone and joint MRI+PET prognostic accuracy for MCI → AD.	- Needs longitudinal MRI and PET for training; limited availability of synchronous multimodal follow-ups. - Focused on amyloid PET; extension to other tracers (e.g., tau PET) is untested but would be valuable.

**Table 2:** Summary of key dataset properties.

Dataset	Subjects	Diagnostic Categories	Imaging Modalities	Longitudinal	Intended Use
ADNI	>2000	3 (CN, MCI, AD)	MRI; PET (amyloid, FDG, tau); CSF, etc.	Yes—multi-year	Diagnosis and Prognosis
OASIS-3	~1100	2–3 (CN, MCI/AD)	MRI (T1, fMRI, DTI, etc.); PET (amyloid)	Yes—up to 15 yrs	Diagnosis and Prognosis
AIBL	2359 (expanded)	3 (CN, MCI, AD)	MRI; PET (amyloid)	Yes—18 mo intervals	Diagnosis and Prognosis
BioFINDER	~2000	3 (CN, MCI, AD)	MRI; PET (amyloid and tau); CSF	Yes—2–5+ yrs	Diagnosis and Prognosis
TADPOLE	219 (ADNI subset)	3 (CN, MCI, AD)	MRI; PET; CSF; clinical tests	Yes—predict 5 yrs	Prognosis (Challenge)
MIRIAD	69	2 (AD, CN)	MRI (T1-weighted)	Yes—up to 2 yrs	Reliability (Atrophy)

**Table 3:** ADNI binary diagnosis (AD vs. CN): comparison of the proposed framework with state-of-the-art methods.

Method	BACC (%)	Precision (%)	Specificity (%)	AUC
ISBI’23	87.5	88.2	88.0	0.90
AnatCL	80.5	82.0	81.0	0.88
DiaMond’25	92.4	93.0	92.8	0.95
SMoCo	82.0	83.1	82.5	0.89
Proposed	93.0	93.6	93.2	0.96

**Table 4:** OASIS-3 diagnosis (ADNI → OASIS-3 transfer): generalization across sites.

Method	BACC (%)	Precision (%)	Specificity (%)	AUC
AnatCL	75.9	76.0	76.5	0.85
DiaMond’25	77.0	77.3	77.1	0.86
ISBI’23	76.5	76.6	76.8	0.85
SMoCo	74.0	74.5	74.8	0.83
Proposed	78.0	78.2	78.4	0.87

**Table 5:** AIBL diagnosis (ADNI → AIBL transfer): performance on a demographically distinct cohort.

Method	BACC (%)	Precision (%)	Specificity (%)	AUC
AnatCL	74.5	75.1	75.2	0.82
DiaMond’25	76.0	76.4	76.3	0.84
ISBI’23	75.0	75.3	75.5	0.83
SMoCo	73.5	74.0	74.2	0.81
Proposed	77.5	77.8	77.9	0.85

**Table 6:** BioFINDER: AD diagnosis and ATN biomarker prediction.

Task	Method	BACC (%)	Precision (%)	Specificity (%)	AUC	ECE (%)
AD vs. CN	AnatCL	74.5	75.1	75.2	0.82	7.2
	DiaMond’25	76.0	76.4	76.3	0.84	7.1
	ISBI’23	75.0	75.3	75.5	0.83	6.2
	SMoCo	73.5	74.0	74.2	0.81	7.8
	Proposed	77.5	77.8	77.9	0.85	5.9
Amyloid (A+/−)	Radiology’23	79.0	78.7	79.1	0.79	7.5
	Proposed	83.0	83.1	83.3	0.83	6.2
Tau (T+/−)	Radiology’23	73.0	72.7	73.1	0.73	8.0
	Proposed	75.0	75.1	75.2	0.75	7.1
Neurodegeneration (N+/−)	Radiology’23	86.0	85.8	86.1	0.86	6.9
	Proposed	85.5	85.3	85.7	0.86	6.8

**Table 7:** TADPOLE prognosis (MCI → AD conversion): longitudinal prediction performance.

Method	BACC (%)	Precision (%)	Specificity (%)	AUC	C-Index	tdAUC@36m	IBS
SMoCo	81.0	81.0	81.5	0.82	0.79	0.79	0.18
ISBI’23	80.0	80.3	80.2	0.81	0.78	0.78	0.19
AnatCL	79.5	79.7	79.8	0.80	0.78	0.77	0.19
DiaMond’25	82.5	82.7	82.6	0.84	0.80	0.82	0.17
Proposed	84.0	84.2	84.3	0.85	0.82	0.82	0.16

**Table 8:** MIRIAD: reliability of longitudinal atrophy progression prediction.

Method	BACC (%)	Precision (%)	Specificity (%)	AUC	Test–Retest ICC	Atrophy Sensitivity (%)
ISBI’23	83.0	83.2	83.5	0.84	0.87	72.0
AnatCL	82.0	82.3	82.5	0.83	0.86	70.5
DiaMond’25	85.0	85.3	85.4	0.86	0.89	74.0
SMoCo	81.5	81.8	81.6	0.83	0.85	69.8
Proposed	87.0	87.2	87.4	0.88	0.91	76.5

## Data Availability

The data presented in this study are openly available in [27,28,29,30,31,32,33,34,35,36,37].

## References

[1] Kwak M.G., Su Y., Chen K., Weidman D., Wu T., Lure F., Li J., Alzheimer’s Disease Neuroimaging Initiative (2023). Self-Supervised Contrastive Learning to Predict the Progression of Alzheimer’s Disease with 3D Amyloid-PET. Bioengineering.

[2] Van Dyck C.H., Swanson C.J., Aisen P., Bateman R.J., Chen C., Gee M., Kanekiyo M., Li D., Reyderman L., Cohen S. (2023). Lecanemab in Early Alzheimer’s Disease. N. Engl. J. Med..

[3] Cummings J., Lee G., Ritter A., Sabbagh M., Zhong K. (2020). Alzheimer’s Disease Drug Development Pipeline: 2020. Alzheimers Dement. Transl. Res. Clin. Interv..

[4] Illakiya T., Ramamurthy K., Siddharth M.V., Mishra R., Udainiya A. (2023). AHANet: Adaptive Hybrid Attention Network for Alzheimer’s Disease Classification Using Brain Magnetic Resonance Imaging. Bioengineering.

[5] Lu P., Hu L., Zhang N., Liang H., Tian T., Lu L. (2022). A Two-Stage Model for Predicting Mild Cognitive Impairment to Alzheimer’s Disease Conversion. Front. Aging Neurosci..

[6] Li Y., Ghahremani M., Wally Y., Wachinger C. DiaMond: Dementia Diagnosis with Multi-Modal Vision Transformers Using MRI and PET. Proceedings of the 2025 IEEE/CVF Winter Conference on Applications of Computer Vision (WACV).

[7] Dukart J., Mueller K., Horstmann A., Barthel H., Möller H.E., Villringer A., Sabri O., Schroeter M.L. (2011). Combined Evaluation of FDG-PET and MRI Improves Detection and Differentiation of Dementia. PLoS ONE.

[8] Tran A.T., Tran A.T., Zeevi T., Payabvash S. (2025). Strategies to Improve the Robustness and Generalizability of Deep Learning Segmentation and Classification in Neuroimaging. BioMedInformatics.

[9] Caron M., Touvron H., Misra I., Jégou H., Mairal J., Bojanowski P., Joulin A. Emerging Properties in Self-Supervised Vision Transformers. Proceedings of the 2021 IEEE/CVF International Conference on Computer Vision (ICCV).

[10] Lew C.O., Zhou L., Mazurowski M.A., Doraiswamy P.M., Petrella J.R., Alzheimer’s Disease Neuroimaging Initiative (2023). MRI-Based Deep Learning Assessment of Amyloid, Tau, and Neurodegeneration Biomarker Status across the Alzheimer Disease Spectrum. Radiology.

[11] Dwivedi S., Goel T., Tanveer M., Murugan R., Sharma R. (2022). Multimodal Fusion-Based Deep Learning Network for Effective Diagnosis of Alzheimer’s Disease. IEEE Multimed..

[12] Barbano C.A., Brunello M., Dufumier B., Grangetto M., Alzheimer’s Disease Neuroimaging Initiative (2024). Anatomical Foundation Models for Brain MRIs. arXiv.

[13] Gryshchuk V., Singh D., Teipel S., Dyrba M., ADNI, AIBL, FTLDNI Study Groups (2025). Contrastive Self-Supervised Learning for Neurodegenerative Disorder Classification. Front. Neuroinform..

[14] Kang S., Kim S.W., Seong J.K., Alzheimer’s Disease Neuroimaging Initiative (2024). Disentangling Brain Atrophy Heterogeneity in Alzheimer’s Disease: A Deep Self-Supervised Approach with Interpretable Latent Space. NeuroImage.

[15] Gong H., Wang Z., Huang S., Wang J. (2024). A Simple Self-Supervised Learning Framework with Patch-Based Data Augmentation in Diagnosis of Alzheimer’s Disease. Biomed. Signal Process. Control.

[16] Yan Y., Somer E., Grau V. (2019). Classification of Amyloid PET Images Using Novel Features for Early Diagnosis of Alzheimer’s Disease and Mild Cognitive Impairment Conversion. Nucl. Med. Commun..

[17] Khatri U., Kwon G.R. (2023). Explainable Vision Transformer with Self-Supervised Learning to Predict Alzheimer’s Disease Progression Using 18F-FDG PET. Bioengineering.

[18] Thrasher J., Devkota A., Tafti A.P., Bhattarai B., Gyawali P., Alzheimer’s Disease Neuroimaging Initiative (2024). TE-SSL: Time and Event-Aware Self-Supervised Learning for Alzheimer’s Disease Progression Analysis. arXiv.

[19] Ouyang J., Zhao Q., Adeli E., Peng W., Zaharchuk G., Pohl K.M. (2023). LSOR: Longitudinally-Consistent Self-Organized Representation Learning. Medical Image Computing and Computer-Assisted Intervention (MICCAI 2023).

[20] Jack C.R., Knopman D.S., Jagust W.J., Shaw L.M., Aisen P.S., Weiner M.W., Petersen R.C., Trojanowski J.Q. (2010). Hypothetical Model of Dynamic Biomarkers of the Alzheimer’s Pathological Cascade. Lancet Neurol..

[21] Kwak M.G., Mao L., Zheng Z., Su Y., Lure F., Li J. (2024). A Cross-Modal Mutual Knowledge Distillation Framework for Alzheimer’s Disease Diagnosis: Addressing Incomplete Modalities. medRxiv.

[22] Zhang Y., Sun K., Liu Y., Shen D. Transformer-Based Multimodal Fusion for Early Diagnosis of Alzheimer’s Disease Using Structural MRI and PET. Proceedings of the 2023 IEEE 20th International Symposium on Biomedical Imaging (ISBI).

[23] Kaczmarek E., Szeto J., Nichyporuk B., Arbel T. (2025). SSL-AD: Spatiotemporal Self-Supervised Learning for Generalizability and Adaptability across Alzheimer’s Prediction Tasks and Datasets. arXiv.

[24] Ouyang J., Zhao Q., Adeli E., Zaharchuk G., Pohl K.M. (2024). SOM2LM: Self-Organized Multi-Modal Longitudinal Maps. Medical Image Computing and Computer-Assisted Intervention—MICCAI 2024.

[25] Yin Y., Jin W., Bai J., Liu R., Zhen H. SMIL-DeiT: Multiple Instance Learning and Self-Supervised Vision Transformer Network for Early Alzheimer’s Disease Classification. Proceedings of the 2022 International Joint Conference on Neural Networks (IJCNN).

[26] Fedorov A., Geenjaar E., Wu L., Sylvain T., DeRamus T.P., Luck M., Misiura M., Mittapalle G., Hjelm R.D., Plis S.M. (2024). Self-Supervised Multimodal Learning for Group Inferences from MRI Data: Discovering Disorder-Relevant Brain Regions and Multimodal Links. NeuroImage.

[27] Wang C., Piao S., Huang Z., Gao Q., Zhang J., Li Y., Shan H., Alzheimer’s Disease Neuroimaging Initiative (2024). Joint Learning Framework of Cross-Modal Synthesis and Diagnosis for Alzheimer’s Disease by Mining Underlying Shared Modality Information. Med. Image Anal..

[28] Weber C.J., Carrillo M.C., Jagust W., Jack C.R., Shaw L.M., Trojanowski J.Q., Saykin A.J., Beckett L.A., Sur C., Rao N.P. (2021). The Worldwide Alzheimer’s Disease Neuroimaging Initiative: ADNI-3 Updates and Global Perspectives. Alzheimers Dement. Transl. Res. Clin. Interv..

[29] LaMontagne P.J., Benzinger T.L., Morris J.C., Keefe S., Hornbeck R., Xiong C., Grant E., Hassenstab J., Moulder K., Vlassenko A.G. (2019). OASIS-3: Longitudinal Neuroimaging, Clinical, and Cognitive Dataset for Normal Aging and Alzheimer Disease. medRxiv.

[30] Leon-Llamas J.L., Villafaina S., Murillo-Garcia A., Gusi N. (2021). Impact of Fibromyalgia in the Hippocampal Subfields Volumes of Women—An MRI Study. Int. J. Environ. Res. Public Health.

[31] Rebsamen M., Rummel C., Reyes M., Wiest R., McKinley R. (2020). Direct Cortical Thickness Estimation Using Deep Learning-Based Anatomy Segmentation and Cortex Parcellation. Hum. Brain Mapp..

[32] Fowler C., Rainey-Smith S.R., Bird S., Bomke J., Bourgeat P., Brown B.M., Burnham S.C., Bush A.I., Chadunow C., Collins S. (2021). Fifteen Years of the Australian Imaging, Biomarkers and Lifestyle (AIBL) Study: Progress and Observations from 2,359 Older Adults Spanning the Spectrum from Cognitive Normality to Alzheimer’s Disease. J. Alzheimers Dis. Rep..

[33] Ellis K.A., Bush A.I., Darby D., De Fazio D., Foster J., Hudson P., Lautenschlager N.T., Lenzo N., Martins R.N., Maruff P. (2009). The Australian Imaging, Biomarkers and Lifestyle (AIBL) Study of Aging: Methodology and Baseline Characteristics of 1112 Individuals Recruited for a Longitudinal Study of Alzheimer’s Disease. Int. Psychogeriatr..

[34] Binette A.P., Smith R., Salvadó G., Tideman P., Glans I., van Westen D., Groot C., Ossenkoppele R., Stomrud E., Parchi P. (2025). Evaluation of the Revised Criteria for Biological and Clinical Staging of Alzheimer Disease. JAMA Neurol..

[35] Ossenkoppele R., Salvadó G., Janelidze S., Pichet Binette A., Bali D., Karlsson L., Palmqvist S., Mattsson-Carlgren N., Stomrud E., Therriault J. (2025). Plasma p-tau217 and Tau-PET Predict Future Cognitive Decline among Cognitively Unimpaired Individuals: Implications for Clinical Trials. Nat. Aging.

[36] Hernandez M., Ramon-Julvez U., Ferraz F., ADNI Consortium (2022). Explainable AI toward Understanding the Performance of the Top Three TADPOLE Challenge Methods in the Forecast of Alzheimer’s Disease Diagnosis. PLoS ONE.

[37] Marinescu R.V., Oxtoby N.P., Young A.L., Bron E.E., Toga A.W., Weiner M.W., Barkhof F., Fox N.C., Eshaghi A., Toni T. (2021). The Alzheimer’s Disease Prediction of Longitudinal Evolution (TADPOLE) Challenge: Results after 1 Year Follow-Up. Mach. Learn. Biomed. Imaging.

[38] Malone I.B., Cash D., Ridgway G.R., MacManus D.G., Ourselin S., Fox N.C., Schott J.M. (2013). MIRIAD—Public Release of a Multiple Time Point Alzheimer’s MR Imaging Dataset. NeuroImage.

